# Human Wavelength Discrimination of Monochromatic Light Explained by Optimal Wavelength Decoding of Light of Unknown Intensity

**DOI:** 10.1371/journal.pone.0019248

**Published:** 2011-05-20

**Authors:** Li Zhaoping, Wilson S. Geisler, Keith A. May

**Affiliations:** 1 Department of Computer Science, University College London, London, United Kingdom; 2 Center for Perceptual Systems, The University of Texas at Austin, Austin, Texas, United States of America; University of Sussex, United Kingdom

## Abstract

We show that human ability to discriminate the wavelength of monochromatic light can be understood as maximum likelihood decoding of the cone absorptions, with a signal processing efficiency that is independent of the wavelength. This work is built on the framework of ideal observer analysis of visual discrimination used in many previous works. A distinctive aspect of our work is that we highlight a perceptual confound that observers should confuse a change in input light wavelength with a change in input intensity. Hence a simple ideal observer model which assumes that an observer has a full knowledge of input intensity should over-estimate human ability in discriminating wavelengths of two inputs of unequal intensity. This confound also makes it difficult to consistently measure human ability in wavelength discrimination by asking observers to distinguish two input colors while matching their brightness. We argue that the best experimental method for reliable measurement of discrimination thresholds is the one of Pokorny and Smith, in which observers only need to distinguish two inputs, regardless of whether they differ in hue or brightness. We mathematically formulate wavelength discrimination under this wavelength-intensity confound and show a good agreement between our theoretical prediction and the behavioral data. Our analysis explains why the discrimination threshold varies with the input wavelength, and shows how sensitively the threshold depends on the relative densities of the three types of cones in the retina (and in particular predict discriminations in dichromats). Our mathematical formulation and solution can be applied to general problems of sensory discrimination when there is a perceptual confound from other sensory feature dimensions.

## Introduction

In a classical wavelength discrimination experiment, the observer views a bipartite field, one half filled with light of a standard wavelength and the other with light of a comparison wavelength. The wavelength of the comparison field is changed in small steps and the observer adjusts the radiance of the comparison field following each change in an attempt to make the two fields perceptually identical. Wavelength discrimination threshold is reached when the observer reports that the two fields always appear different, regardless of the radiance of the comparison [1]. This discrimination threshold in humans is a “w” shaped function of the wavelength of the light: it has a central peak at around wavelength 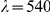 nanometers (nm), minima at 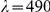 and 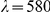 nm, and rises up sharply for 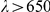 nm and for very short wavelengths[1]; similar results hold for the macaque monkey and presumably other old world primates[2].

This work aims to see if human monochromatic light discrimination thresholds can be understood as optimal decoding of the sensory input using the information available in the cones, regardless of the specific neural mechanisms involved. In particular, we derive and evaluate a photon noise limited ideal observer that performs wavelength discrimination based on the numbers of photons absorbed in the three classes of cone. It is well known that human performance does not approach that of a photon noise limited ideal observer[3], [ 4], [ 5], [ 6], and thus our primary aim here is to determine how well the shape of the human wavelength discrimination function is explained by the ideal observer, regardless of its overall amplitude. If the shape were perfectly explained, then it would imply that the neural mechanisms following the cones are equally efficient for different wavelengths.

Wavelength discrimination of monochromatic lights is one of the visual tasks most suited to ideal observer analysis for the following reasons. Input sampling by the photoreceptors is among the best quantitatively understood process along the visual processing pathway. In particular, the wavelength sensitivities of cones are known, and the stochastic nature of the cone absorption levels can be described by Poisson distributions of absorption levels. The discrimination task is simple because it involves purely chromatic discrimination, so the spatial and temporal aspects of the inputs can be ignored or absorbed by the scale for the total input intensity. Therefore, total cone absorptions by the excited cones can lead to sufficient statistics for analysing the consequent decoding and its uncertainty of the input stimulus.

There have been many previous studies using ideal observer analysis to understand human visual performance[7], [ 3], [ 4], [ 5], [ 8], [ 6]. Geisler[8] in particular used such an analysis to understand many human discrimination tasks based on cone responses. Among these tasks analyzed is our task of monochromatic light discrimination. His work and the current work are both based on the maximum likelihood method which can be used to optimally estimate or discriminate sensory inputs from their evoked neural responses. These two methods are approximately equivalent in the principle of maximum likelihood discrimination of two stimuli. However, this previous work did not identify an important issue that is essential for fully understanding the behavioral data. This issue is that of a confound in perception of multiple sensory features – in particular, human observers can easily confuse an input color change with an input intensity change when monochromatic lights are the inputs; for example a long wavelength input may appear darker when the input wavelength is increased while input intensity is held fixed. This confusion reduces human ability in hue discrimination when observers do not have the full knowledge of input intensities. To fully account for the behavioral data, this confound should be formulated explicitly in the ideal observer analysis.

The current work presents an augmented formulation of the ideal observer analysis to address sensory discrimination under a perceptual confound, and applies it to wavelength discrimination behavior. The sensory input includes both sensory feature dimensions: one is the input wavelength dimension whose discrimination is of interest, and the other is the input intensity dimension which interferes or interacts with wavelength discrimination through the perceptual confound and the experimental methods used. Our mathematical formulation of this problem of sensory discrimination under perceptual confound is general. While it is applied specifically to the wavelength discrimination problem in this paper, it can also be applied elsewhere. It will enable us to identify experimental methods which can provide more reliable measurments of the discrimination performance. From our formulation, we derive how the threshold is related to the cones' wavelength sensitivities and the input light intensity, illustrate how sensitively the predictions depend on the relative densities of the three types of cones in the retina, and analyze why the discrimination threshold varies with the input wavelength in the ways observed. We show that our theoretical predictions from the augmented ideal observer analysis to accommodate the perceptual confound can give a better account of the behavioral data. Furthermore, we show how different sizes of stimuli used by different experiments may explain their different patterns of results. A preliminary report about this work has been presented elsewhere[9].

## Methods

### The spectral sensitivities of the cones

Let there be three types of cone 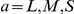, which are most sensitive to long, medium, and short wavelengths respectively (they are sometimes called red, green, and blue cones). They have tuning curves 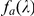, such that the average cone absorption of a single cone 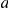 to a monochromatic light of intensity 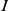 at wavelength 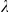 is 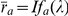. If 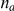 cones of type 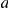 are excited by a uniform patch of light, then the essential quantities for determining input color, regardless of the spatial shape of the input patch, are the total responses from each of the three cone types. For the task of color discrimination, it is equivalent to view the 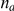 cones of type 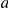 collectively as a single giant cone with sensitivity 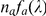, for this giant cone's sensitivity provides a sufficient statistic for the task (i.e., this sensitivity provides all the information relevant to the task) such that viewing individual cones separately does not provide any additional useful information for the task. The all-important ratios 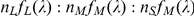 depend on both the relative densities and the relative sensitivities of the different cone types.

According to various experimental data on the responses from and light absorption by cones [10, 11, 12], 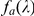 for different cones should peak to the same peak value, if one ignores the pre-receptor absorption by the ocular media. We denote this normalized spectral sensitivity as 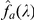, and will call it the cone fundamental. However, pre-receptor absorption of the input lights by the ocular media makes 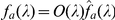 where 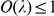 is the pre-receptor absorption factor. Let 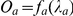, where 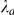 is the wavelength where 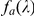 peaks; then 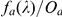 should correspond to the behaviorally measured (normalized) cone fundamental, and for notation simplicity we still denote it as 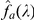 and thus 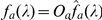. Meanwhile, assuming that 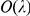 does not change as quickly as 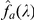 with 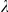 near 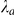, then 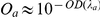 where 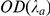 is the optical density of the pre-receptor ocular media at wavelength 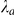.

In our analysis later, we will include the cone density factor 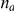 and use the notation 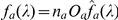. Furthermore, we normalize 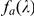 such that Max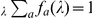. Given these normalizations, the total photon absorptions of the cones will also scale with the size of the input light field (which determines the total number of cones for each cone type) and the effective input integration time by the viewing of the observers. These scale factors will be absorbed into the input intensity parameter 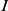, which also scales with the input radiance. We will see later that, given 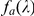, the shape of the curve relating the discrimination threshold to wavelength is completely determined by the optimal decoding, and the parameter 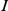 merely scales the threshold.

**Figure 1:**
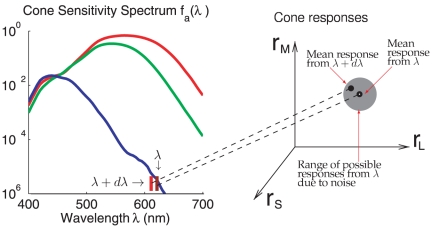
Illustrations of noisy encoding of monochromatic inputs by the cone responses. On the left is the cone spectral sensitivity 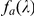 (with 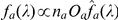, where 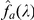
s are derived from the Smith and Pokorny cone fundamentals[15], the cone density ratio is 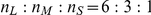, the pre-receptor light transmission factors 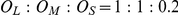, and Max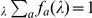). A monochromatic input of wavelength 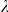 evokes response 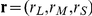 from the three cones, L, M, and S. Due to input noise, there is a range of possible responses 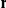 from this input. If the mean response to a monochromatic input of nearby wavelength 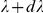 is one of the typical responses within this range of responses 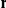 to input 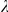, then it will be difficult to perceptually distinguish the input 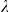 from input 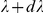.

As our illustrative starting point, we approximate 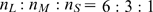 and 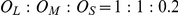. These numerical values arise from the following considerations. Firstly, various sources suggest that S cones are almost absent within 0.3 deg from the center of fovea but their contribution to the total cone density rises and peaks to 15% around 1 deg from the center[13] and approaches 7–10% in the periphery[13], [ 14]. Meanwhile, the Pokorny and Smith data[1] were from experiments using a centrally viewed 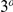 disc containing the bipartite field of color inputs. We combine this information to assume that the S cones contribute 10% to all cones excited by the Pokorny and Smith stimuli. Secondly, various sources suggest that L cones are about twice as numerous as the M cones[14], we hence assume that L and M cones contribute 60% and 30%, respectively, of all the excited cones by the stimuli. This gives us 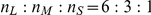. Thirdly, the optical density of the pre-receptor ocular media is almost constant in the medium and long wavelength region, giving 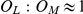, but rises with decreasing 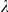 by 0.7 log units when 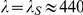 nm[14], giving 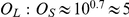. Additionally, although the cone fundamentals 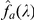 from various literature sources are similar, we use those from Smith and Pokorny[15] (obtained from the CVRL website (http://www.cvrl.org) by Andrew Stockman), since we will be fitting their wavelength discrimination data[1]. Combining the considerations above gives 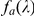 as shown in Fig. 1. It turns out that these 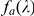
's are not far from those by Vos and Walraven[16], who made 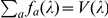 where 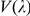 is the luminous efficiency function, a measure of the visual effectiveness of lights at different wavelengths for luminosity, normalized such that the maximum value of 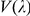 is 1, i.e., Max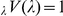. The biggest discrepancy between the two sets of 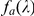
's is that the S cone contribution is weaker in Vos and Walraven's composition[16] than in ours. This is not too surprising, as although the relative contributions by different cone types to luminosity perception are not necessarily the same as their relative contributions to color perception, they should be related or quite close to each other, except that S cones may contribute to the luminosity perception less than suggested by their density[17]. Our analysis and conclusions do not depend sensitively on our actual approximation for 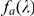. We will later explore how our results vary quantitatively when we use other choices for the ratio 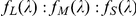. This ratio depends on cone densities and the optical density of the pre-receptor ocular media, which both vary substantially between observers (e.g., by up to one log unit in optical density[14]). This ratio 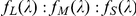 also depends on the cone spectral sensitivities, which do not vary as substantially between observers but different literature sources provide slightly different quantitative values for them.

### Stochastic cone absorptions in response to monochromatic light

In this paper, we only consider monochromatic inputs. Hence, we describe our input stimulus by 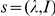, a vector of two parameters, 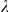 and 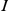, for the wavelength and intensity of the input light. The actual cone absorption 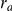 for cone 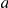 is stochastic following a Poisson distribution with a mean 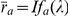


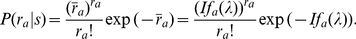
(1)


Sometimes we also call 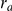 the response of the cone to the input light. The population response 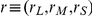 has the probability
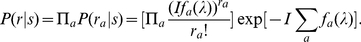
(2)


Fig. 1 shows how an input of particular wavelength could give rise to many possible responses in the three dimensional space 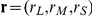 near the mean response 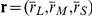.

### Maximum likelihood decoding

Given the responses 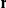, one can decode the input stimulus 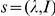 from the conditional probability 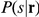 (of 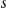 given 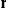) by finding the 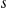 that makes 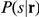 maximum or large. So the most likely input to evoke 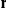 is the one that maximizes 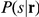. By Bayes's formula, we have 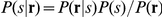 where 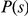 is the prior probability of input 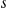 and 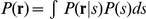. When the prior probability 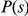 is constant so that it does not favour one 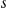 over another, then 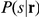 varies with 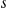 only through 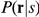, i.e.,
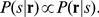
(3)


Therefore, the input 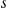 for responses 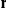 can be found by maximizing 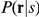. As 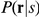 is also called the likelihood of 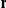 given 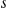, decoding by maximizing 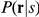 is called maximum likelihood decoding. We will use this method to understand wavelength discrimination.

### Decoding for input wavelength when input intensity is known and fixed

When input intensity 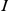 is known and fixed, knowing the response 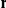 enables us to estimate the input wavelength 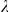 using maximum likelihood decoding. We call this the simple model of optimal input wavelength estimation, in the sense that we are not considering the variation of 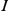 (as in experimental procedure of Pokorny and Smith[1]) in decoding. With a flat prior expectation that 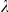 could be any value (within the visible light spectrum), the best estimate 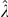 for the input 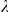 is the one that maximizes the probability 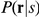 or equivalently its natural logarithm, 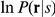,
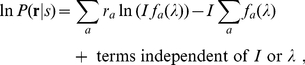
(4)which we call the log likelihood.

The best estimate 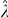 is the value of 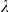 satisfying
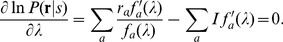
(5)


In a special case, if 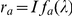 under input 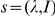 for all three cones (i.e., the response of each cone type is exactly equal to the mean absorption), then 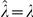 is the value satisfying the above equation. In general, there is no 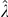 to make 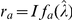 exactly for all three cones simultaneously, but one can still find a 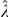 to satisfy the equation above. In any case, given an input wavelength 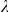, different responses 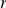 will lead to different estimates 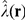; most of them will be near to but not equal to the actual input wavelength 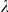. So if two different input wavelengths 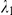 and 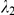 are similar enough, the estimated wavelengths 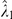 and 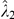 may appear to be drawn from the same probability distribution. In such a case, these two input wavelengths would appear perceptually indiscriminable, or within the discrimination threshold; see Fig. 1.

With strong enough responses 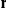 (effectively responses collected from enough cones and sufficiently many captured photons), it is known that the variance of these maximum likelihood decoded 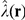 for a given input 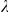 should approach[18]
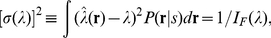
(6)


where 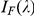 is the Fisher information defined as
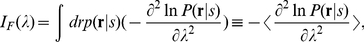
(7)


where 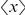 denotes average 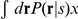 of 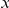 over 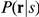. Since
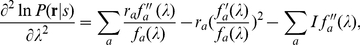
(8)


and 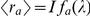, we have
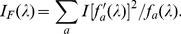
(9)


As 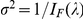, a larger Fisher information gives a smaller estimation error 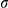. This estimation error can be expressed as
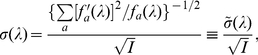
(10)


in which 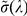 does not depend on intensity 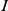.

**Figure 2:**
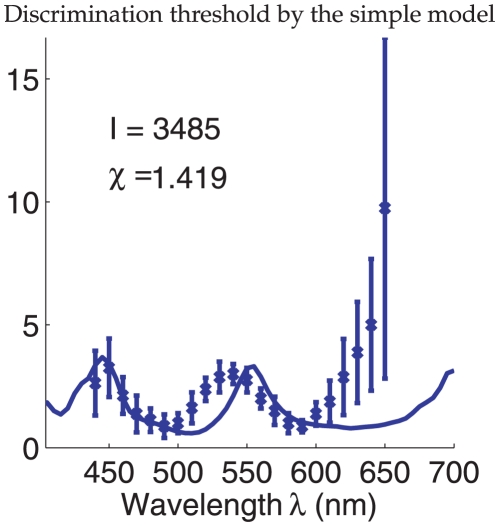
Wavelength discrimination assuming input intensity 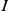 is fixed and known during color matching. It is by maximum likelihood decoding of the cone responses 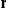 using the simple model. The solid curve plots the discrimination threshold 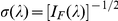 as a function of 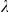 from the model. The data points with error bars are the mean 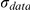 and the standard deviation 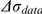 of the discrimination thresholds of the four observers of Pokorny and Smith[1]. In fitting the model to the data, 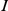 is chosen such that the quantity 
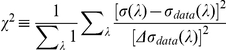
 is minimized.

The estimation error 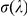 is identified here as the discrimination threshold, as it characterizes the uncertainty of the perceived wavelength. Fig. 2 shows this threshold 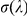 as a function of 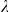, together with the experimentally observed threshold 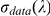 from Pokorny and Smith[1]. Let 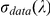 and 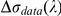 be the mean and the standard deviation of the wavelength discrimination thresholds of the four observers in Pokorny and Smith[1]. The input intensity 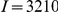 in Fig. 2 is chosen as the one that minimizes the average square difference:
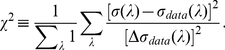
(11)


The 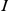 that minimizes 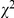 is the one that gives 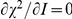, leading to (since 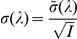)
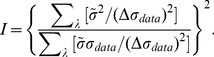
(12)


One can see that the model prediction greatly underestimates the threshold for long wavelengths 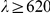 nm. Also, the peak location near 550 nm is not quite right. This best fit gives 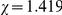, indiciating that for most data points, the model predicts a threshold which departs from the data by more than a standard deviation of the data point.

The poor fit of the simple model arises because of the following. In Pokorny and Smith's experiment, observers adjusted the intensity 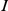 of the comparison input field with wavelength 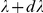 to make it look as perceptually indistinguishable as possible from the standard input field which has input wavelength 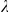. This adjustment makes the comparison and standard input fields look indistinguishable until 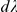 is too large, and the wavelength discrimination threshold is defined as the 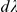 when this matching between the two fields starts to become impossible, so the comparison field is perceptually discriminable from the standard field no matter how observers adjust the intensity 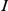. If the observers somehow had the full knowledge of the intensities 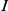 in both fields, they should in principle still be able to decode and thus discriminate the wavelength to roughly the same accuracy as predicted by the simple model when the intensity is held fixed and identical in the two fields. The reason the predictions overestimate the human accuracy is because one should not assume that the observers know the intensities 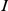, which also have to be decoded from the same sensory stimuli used to decode the wavelength. To explain the experimental data, our model should let 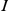 be unknown and changeable rather than known and fixed. We call this the full model (rather than the simple model) of optimal wavelength estimation, and this model is explained next.

### Sensory discrimination under perceptual confound – wavelength discrimination when input intensity is not fixed

Wavelength discrimination when input intensity is not fixed is just one example of a general problem of sensory discrimination under perceptual confound: sensory discrimination along one sensory feature dimension when neural responses are also affected by feature changes in another feature dimension. In the wavelength discrimination case, the two feature dimensions are input light wavelength 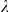 and input intensity 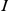. Here, we formulate this problem in general, and it will be clear that our result in equation (20) is general and not specific to our example of monochromatic wavelength discrimination. Meanwhile, we will use our wavelength discrimination problem as an example to illustrate this general result.

Let the sensory input be 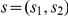, where 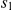 and 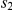 are feature values in the two feature dimensions, e.g., 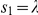 and 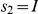. Let 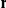 be the neural responses evoked by 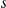 with probability 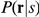. The maximum likelihood estimation 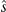 of 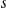 from 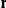 can be arrived at by finding the solution to
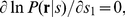
(13)

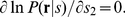
(14)


The estimation error is
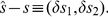
(15)


This error depends on the specific response 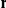 in each trial. Over many trials, these two dimensional errors 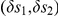 have a covariance, generalizing from the simple 1-dimensional case above, given by
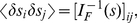
(16)


where 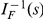 is the matrix inverse of the Fisher information matrix
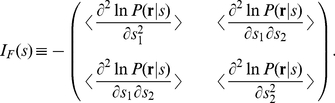
(17)


We note that, when 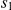 is 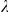 in our example, the matrix element 
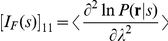
 is exactly the Fisher information we had in our simple model of wavelength discrimination.

Let the 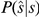 be the probability of obtaining the maximum likelihood estimate 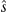 when the true input is 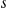. Since the estimation error 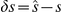 has the covariance structure in equation (16), we can approximate 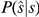 as
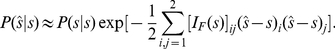
(18)


Note that this approximation makes the error 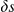 have zero mean and gives the correct error covariance.

Now the threshold to discriminate 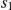 while 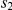 is not fixed is the largest 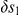 value that can be obtained to maintain 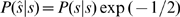, i.e., to give
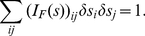
(19)


**Figure 3:**
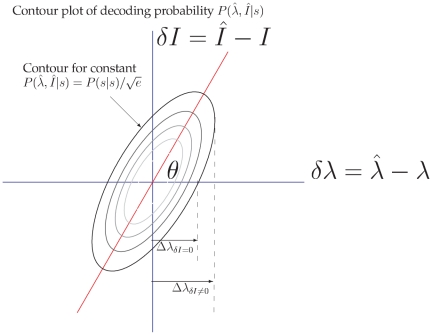
Illustration of 2D decoding in the full model. Given the true input 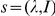, 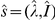 is the estimated input parameters. This plot illustrates the conditional probability 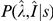, since a given 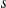 may evoke different responses 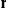 leading to different 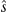. The wavelength discrimination threshold 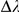 when 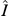 is allowed to deviate from 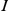 is larger than otherwise.

Applying the above to the example of wavelength discrimination, the threshold for wavelength 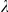 discrimination while 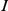 is not fixed is the largest 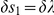 value that can be obtained to maintain 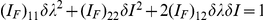, a particular example of equation (19). This can be illustrated in Fig. 3. This figure shows the contour plot of the posterior probability 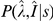. This probability peaks at the origin 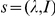 of the coordinates in this plot. As deviation 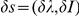 of 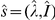 from 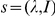 increases, the probability 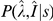 decreases, as indicated by the contours of probabilities, with larger, darker, contours indicating smaller probabilities. When 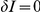, the largest 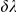 to make 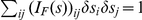 is 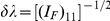, the color discrimination threshold in the simple model and indicated by 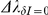 in Fig. 3. If 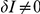, then the largest wavelength deviation 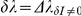 on the contour 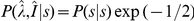 should be larger, as indicated in the figure. This condition of 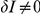 means that the decoding system assumes that 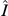 can be different from the default 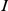, i.e., the intensity of the comparison field can be different from the intensity of the standard field in the color matching.

We can show (detailed derivation in the next subsection after equation (28)) that the discrimination threshold for feature 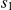 when input feature 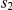 is not fixed (e.g., wavelength discrimination threshold at wavelength 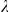 when input intensity 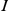 is not fixed) is
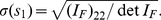
(20)


In particular, 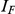 in our wavelength discrimination problem is
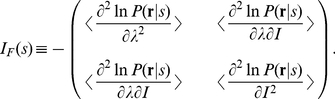
(21)


Since we have
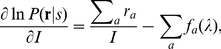
(22)

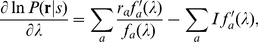
(23)


and
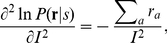
(24)

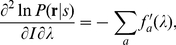
(25)

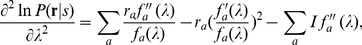
(26)


then, given 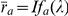, we have
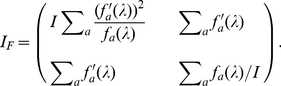
(27)


Plugging the above into equation (20) we have
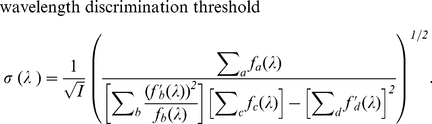
(28)


Again, this threshold can be writen as 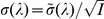. This predicts precisely how wavelength discrimination threshold should vary with wavelength 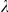, and that it should scale with 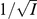 as in the simple model. Like the simple model, the full model only has one free parameter, 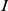.

### Mathematical proof of equation (20)

For matrix 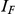, let us denote its normalized eigenvectors as 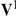 and 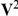, with corresponding eigenvalues 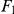 and 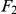. Note that the two eigenvectors 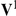 and 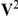 are orthogonal to each other, since 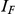 is a symmetric matrix, so any 2 dimensional vector 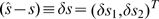 (where the superscript 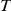 denotes transpose) can be expanded in their basis as 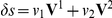 with coefficients 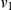 and 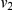 respectively. Then 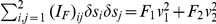 due to the invariance of this quantity to the bases used. Note that since 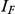 is positive definite, 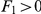 and 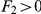. Defining 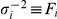, we have
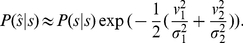
(29)


Analogous to the 1-d case, we find the discrimination threshold by looking at the 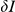 vs. 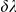 curve such that 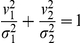, and find the largest 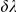 on this curve, and this largest 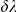 should be the discrmination threshold.

One can always find a parameter 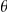 (see Fig. 3), such that the eigenvectors are
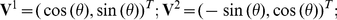
(30)


One notes that the dot product 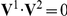. Then we have
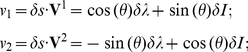
(31)


From these we can solve for 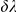 in terms of 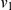 and 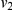 as
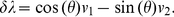
(32)


The values of 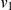 and 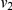 on the curve 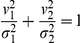 can be described by a parameter 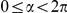 such that
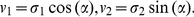
(33)


Hence, we can write 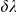 as a function of 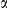 as
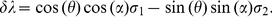
(34)


The largest 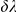 is when 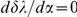, giving
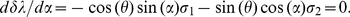
(35)


The above is satisfied when
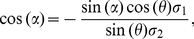
(36)and
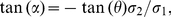
(37)and since 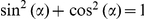, and 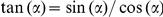; then we have
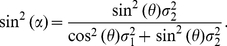
(38)


Plug equation (36) to equation (34), writing 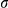 for this extreme 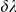 (the discrimination threshold) when 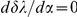, we have
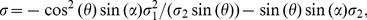
(39)or
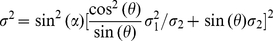
(40)

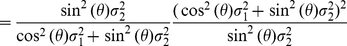
(41)

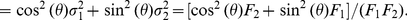
(42)


Noting that, as properties of eigenvectors 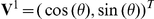, and 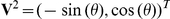,
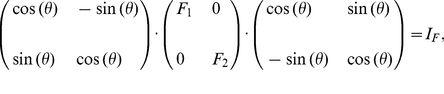
(43)we have, equating 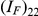 on the right hand side of the equation to that in the left hand side
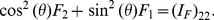
(44)


Also noting that 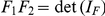, the determinant of the 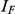 matrix, we have
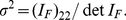
(45)


## Results

**Figure 4:**
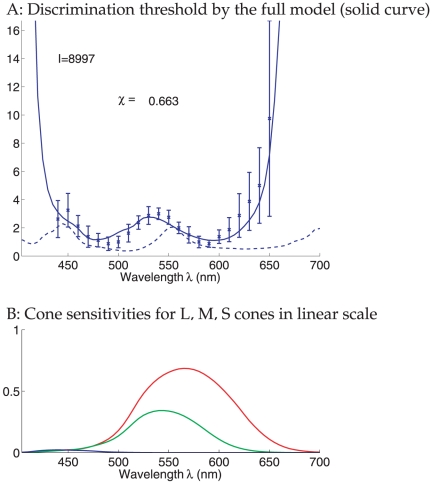
Wavelength discrimination under input intensity confound. A: Wavelength discrimination by maximum likelihood decoding of cone inputs using the full model, assuming that the color matching is done by adjusting both the input intensity 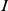 and wavelength 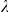 of the comparison field. The solid curve shows the results from the full model. The parameter 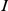 (of the standard field) is chosen such that the quantity 
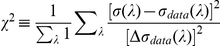
 is minimized. The dashed curve shows the results from the simple model using this same input intensity 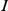. The data points with error bars are the mean 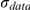 and the standard deviation 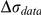 of the discrimination thresholds of the four observers of Pokorny and Smith[1]. B: cone sensitivities plotted on a linear scale.

Figure 4 illustrates the full model's predicted threshold (in equation (28)) fitted to the data. It uses the optimal 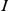, as in equation (12), such that the summed squared difference (as in equation (11)) between the predicted and observed thresholds is minimized. The fitting quality is much better than that by the simple model. In particular, with 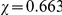, the predicted threshold is within the standard deviation of experimental data for most data points. As in the data, the predicted threshold rises sharply as 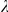 approaches the ends of the spectrum.

### The wavelength-intensity confound and the divergence of threshold near the red and blue ends of the spectrum

The thresholds predicted by the full and simple models differ most towards the red and blue ends of the spectrum. This is because only one cone type can be substantially activated at the spectrum ends, making the system practically color blind, just like in scotopic vision when only the rods are active. For example, in the red end of the spectrum when the M and S cones are almost silent, an increase in 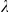, i.e., 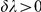, weakens the L cone response 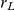, i.e, 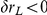. The simple model uses 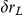 for wavelength discrimination by attributing it to 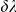 with the relationship 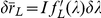. The full model however sees this 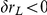 as equally attributable to a reduced input 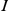, i.e., 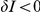, with 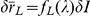, making it hard to distinguish whether the input gets redder or darker. This wavelength-intensity confound for the same 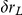 makes wavelength discrimination difficult. In the procedure of the Pokorny and Smith experiment[1], it means that an increase in 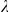 can be easily compensated by an increase in 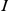, making the threshold large.

The wavelength-intensity confound is present generally even when all cone types are substantially activated. Let 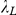, 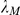, and 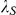, with 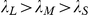, be the preferred wavelengths of the L, M, and S cones respectively. This confound is stronger when 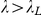 or 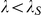, when the predictions from the simple and full models differ most (see Fig. 4. In these wavelength regions, a change 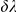 causes response change 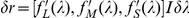, which either simultaneously increases or simultaneously decreases the responses from all cone types, just like the response change 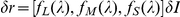 caused by an intensity change 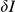. Although a 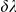 slightly changes the ratio 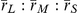 while a 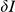 does not, the difference between the 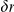 caused by 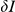 and the 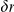 caused by 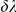 could be submerged under noise such that the two causes are perceptually indistinguishable.

This confound is weaker when 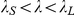, when a wavelength change 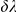 will raise responses from some cone types while lowering responses from other cone types. In this case, a 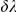 cannot be easily compensated for by an 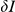, which raises or lowers the responses from all cone types simultaneously. Hence, the simple and full model predict similar thresholds, particularly when 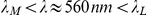 is in between the preferred wavelengths of the two most numerous cone types, L and M. For 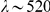 nm, the S cones are still insensitive, while both the L and M cones prefer larger 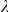, and the confound is again significant, causing a substantial difference in the predicted thresholds from the simple and the full models. This is because a 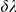 increases or decreases the responses from the L and M cones simultaneously (while affecting the S cone response relatively little), and can be easily compensated for by a 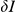.

### Implications of the wavelength-intensity confound on the experimental procedures and on the stability of the threshold measurements

The wavelength-intensity confound, especially when 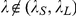, means that there can be problems with some experimental methods used to measure wavelength discrimination threshold. In many such experiments (e.g., [19], [ 20]), the procedure requires adjusting the intensity of the comparison field such that the brightnesses of the two fields match. The confound means that, when observers see a difference between the two fields, it is not easy to tell whether it is a brightness difference or a hue difference. This is a known difficulty noted in the accompanying discussions of Wright and Pitt's paper by fellow color vision scientists (pages 469–473 of [20]). Supposedly, the threshold is the smallest wavelength difference between the two fields when observers deem the two fields to differ in hue but not in brightness. However, whether the observers judge some perceptual difference to be a brightness or hue difference is likely to be dependent on the following factors: observers' internal criteria based on their expectations or biases, specific task instructions given by the experimenters, and perhaps even the visual environment around the experimental set up. These factors cannot be predicted straightforwardly from our optimal decoding theory, and could also cause variabilities between data from different observers and from different laboratories.

The procedure used by Pokorny and Smith[1] differs from the procedure above. They ask the observers to adjust the intensity of the comparison field until the two fields match in both hue and brightness, and the threshold is the smallest wavelength difference when this match is impossible by any intensity adjustment. This procedure does not require observers to decide whether any perceptual difference is due to brightness or hue, as they simply need to judge whether the two fields differ or not. This makes the threshold data more stable. Therefore, we do not intend to compare our theoretical prediction with data other than those by Pokorny and Smith[1].

### The effect of the cone densities and pre-receptor light transmission on wavelength discrimination

It is clear from the analysis that the discrimination threshold depends on the relative sensitivities 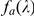 for different cone types 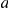. Since our 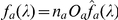 scales with the relative cone density 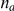 and the relative pre-receptor transmission factor 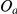 for each cone 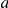, 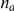 and 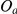 should affect discrimination. We remind ourselves that the cone fundamental 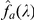 for all cones 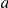 have the same peak value Max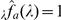, and we have the normalization Max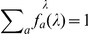. Let us denote 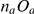 by 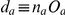, which could be understood as the effective cone density for cone type 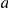. We can rewrite the threshold in equation (28) as
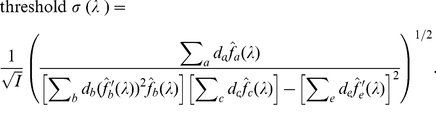
(46)


So 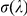 at any particular wavelength 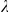 scales roughly with 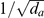 for the cone type 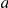 that dominates at 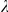. For example, increasing the fraction 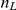 of the L cones among all cones would relatively lower the discrimination threshold near the red end of the spectrum, and increasing light absorption by the pre-receptor ocular media near the short wavelength region would decrease 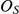 and thus raise the threshold near the blue end of the spectrum.

**Figure 5:**
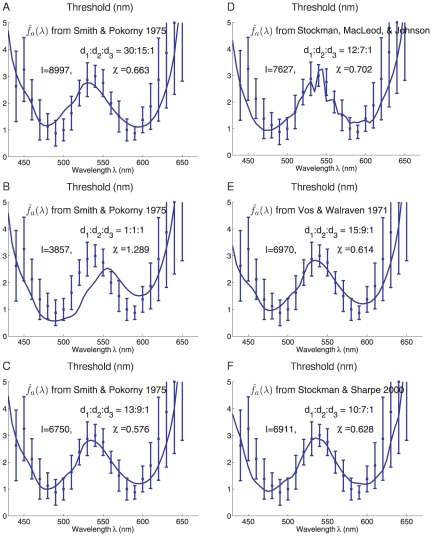
Variations of the model predictions due to variations in the cone fundamentals, cone densities, and pre-receptor transmission. The 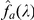 is normalized to the same peak value Max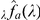, the cone factor 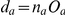 combines the cone density 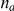 and pre-receptor transmission factor 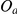, to determine the cone sensitivity 
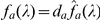
, with normalizations Max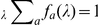. Each plot is like Fig. 4A, having a full model predicted threshold with an optimal 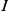. Each is labeled with the literature source for 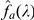 and the 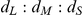 used. A–C have the Smith and Pokorny cone fundamentals[15] with different 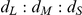. A is a modified plot of Fig. 4A. C–F show the best predictions (the 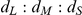 that minimizes 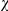) for four different cone fundamentals. Only integer values of 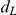, 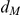, and 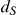 are used (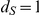 in all cases).

Fig. 5A–C shows the predictions using Smith and Pokorny cone fundamentals[15] 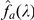 but with different settings for 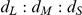. Fig. 5A is a replot of Fig. 4 with different scales on the axes. Its 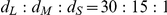 arises from our estimated 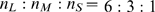 and 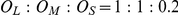 from experimental data[13], [ 14]. We note that its worst predictions are near wavelength 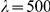 nm, which is in the region where S cones' sensitivity 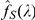 has large slopes 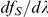 and hence a high sensitivity to wavelength changes. Fig. 5B has 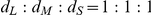 which could be seen as a situation when all cones have the same density and pre-receptor optical transmission. It raises the relative density for the S cones way over the physiological reality, and slightly raises the relative density of the M cones over the L cones. Consequently, it vastly over-estimates the discrimination sensitivities near the region 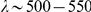 nm, in the domain of the S and M cone contribution. As a result, it gives a 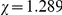 that is substantially worse than the 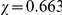 in Fig. 5A. Fig. 5C has a 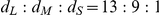 ratio that minimizes 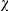, such that the predicted thresholds best agree with experimental data. This 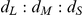 ratio is obtained by exhaustively searching all integer values of 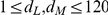 with 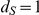 held fixed. With 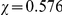, almost all the data points are within a standard deviation from the predicted values. Compared with Fig. 5A, Fig. 5C raises the weights for the S cones (and slightly for the M cones), but not as dramatically as Fig. 5B does. Hence, it corrects the worst predictions in Fig. 5A near the 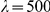 nm region without overshooting the correction.

Fig. 5D–F show the best predicted thresholds like Fig. 5C by three other cone fundamentals 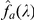 obtained from different sources in the literature: [21], [16], and [22] (see Andrew Stockman's webpage http://www.cvrl.org/). Compared with the predictions when using the Smith and Pokorny cone fundamentals[15] in Fig. 5C, their best predicted 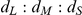 ratios are similar, and so are their goodness of fit 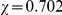, 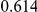, and 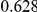, which are only slightly worse than 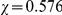 in Fig. 5C. This finding is not so surprising, as the cone fundamentals from different literature sources are similar to each other. Meanwhile, it may not be a coincidence that the cone fundamentals of Smith and Pokorny[15] best fitted the wavelength discrimination data obtained by them. It is likely that different researchers have different research styles and experimental procedures and hence different sets of experimental data obtained by the same style are more likely to be consistent with each other.

### The importance of the S cone minority

Experimental data for wavelength discrimination for 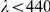 nm are scarse and very variable. These may be caused by many factors, including the large inter-subject variabilities (e.g., in cone densities and optical density of the ocular media) in that wavelength region, the difficulties of delivering stimulus in the short wavelength region, where light absorption by ocular media is dramatic[14], and, as discussed above, the wavelength-intensity confound makes some experimental procedures problematic in that wavelength region. However, Bedford & Wyszecki[19] reported that, as threshold rises with decreasing 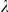 below 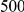 nm, it dips again around 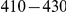 nm before rising sharply. Wright and Pitt reported in 1934[20] a much shallower dip at a slightly larger 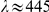 nm. As we argued, a perceptual confound between wavelength and intensity for 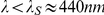, the most preferred wavelength by S cones, should make threshold rise continuously with decreasing 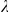 as all three cone types become less and less sensitive. So it may seem puzzling how this dip could arise from our full model, which shows a continuous rise of the threshold as 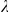 decreases. Bedford and Wyszecki[19] acknowledged and discussed that the presence of this dip was controversial experimentally. In fact, a dip in the very long wavelength region was also seen by earlier studies and was then invalidated by later studies[20], and is no longer seen in modern day data[19], [ 1].

**Figure 6:**
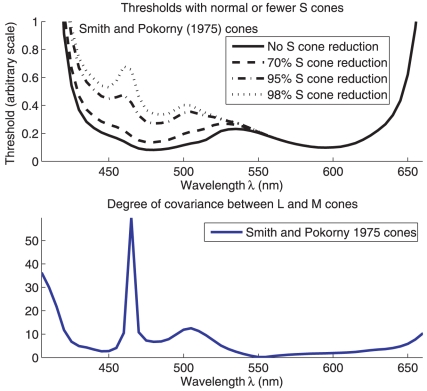
Illustration of how reducing the density of S cones should create a threshold peak near 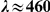
nm. Because the L and M cones have their spectral sensitivity co-vary with each other as 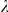 varies near 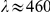 nm, they act as if they are a single cone type around that 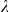. As threshold eventually increases when 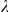 approaches 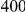 nm, this local threshold peak at 460 nm creates a threshold dip between 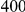 and 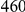 nm.

We suggest that the extra dip near 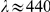 nm may be the side effect of an extra peak in threshold at 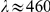 nm caused by too few blue cones involved in some experiments. We note that Bedford and Wyszecki[19] used input bipartite fields that were 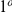 or smaller. This is smaller than the input field 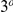 used by Pokorny and Smith[1]. As the density of S cones drops drastically to zero within 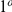 from the center of the fovea[14], there are fewer S cones involved if the central viewing color matching fields are smaller than 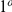. (Note that observers in Bedford and Wyszecki's experiment[19] used free viewing for their task. We consider such free viewing in this attention demanding task as central viewing since gaze follows attention mandatorily in free viewing[23]). If there are no S cones, wavelength discrimination relies on L and M cones only. A close examination of the L and M cone spectral sensitivities reveals that, in a small region of 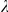 around 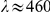 nm, 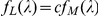 with a scale factor 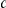 that is almost constant within that region. This means, as 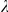 changes in that region, the responses of the L and M cones co-vary almost completely (except for noise) so that they act together as if a single rather than two different cone types. This makes the L+M dichromatic system almost color blind in that local wavelength region, and consequently the discrimination threshold shoots up. This covariance of the two cone types can be seen in the signature 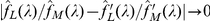, and we can define a degree of co-variance as
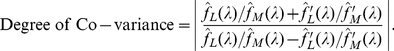
(47)Mathematically, the 2x2 Fisher information matrix 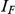 reduces its rank to 1 when both cones have their 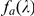 scale with each other, and thus the two dimensional wavelength-intensity input space is collapsed into one by the two redundant cone types acting as one. Fig. 6 illustrates how this Degree of Co-variance between the L and M cones shoots up near 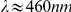, thus giving a peak in threshold around that wavelength when there are too few S cones. The exact location of the peak depends slightly on the 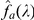 cone fundamentals used, whether it is the[15] cone fundamentals or other cone fundamentals, but this difference is not big. This rise in threshold around 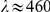 nm can be prevented by having sufficiently many S cones to remove the collapse of dimensionality. The dramatically worse discriminability at 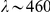 nm with smaller color matching field sizes or in tritanopic dichromats (who lack S cones) has been observed in previous studies ([24], [ 25].

## Discussion

Our maximum likelihood decoding model can explain the experimental data reasonably well. This is based on adjusting a single free parameter, 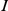, which characterizes the net effect of the radiance of the input light, the effective integration time (within the observer's visual system), and the total area of the input field, etc. Although we did not compute overall quantum efficiencies (i.e., the ratio between the number of photons needed by the ideal and human observers for the same task; [7]), they are undoubtedly quite low (typically they are less than 0.1, [3], [ 4], [ 5]). Nonetheless, the good agreement between the model and data shows that, for wavelength discrimination, the efficiency of human color processing mechanisms is nearly constant over the spectrum (i.e., information is extracted with equal efficiency at all wavelengths).

The best fit between data[1], [ 15] and theoretical prediction suggests that the ratios between effective densities of different cones are 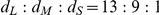. Here, the effective density 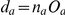 for each cone type 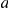 is the actual cone density 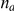 diluted by the pre-receptor optical transmission factor 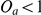. Meanwhile, evidence suggest that on average 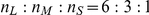 and 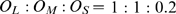 [14], [ 13], giving 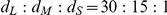. Since variability in human optical density can give up to a factor of 10 difference in 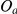, and a difference in human 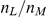 by a factor of 3 seems not unusual[14], our finding of an optimal 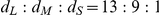 can be seen as within the range of variability of the human quantities.

We analyzed the probable causes of the differences in results across color matching experiments, and how the results could sensitively depend on the experimental procedures and stimulus parameters. It is expected and straightforward to conclude that discrimination threshold should be smaller when color matching is done without adjusting the matching field intensity. Furthermore, we identify that different sizes of the centrally viewed matching fields may cause different findings regarding whether or not there is a dip in discrimination threshold below 450 nm, or a peak around 460 nm. This peak and the resulting dip in particular may arise from small, foveally viewed, fields such that fewer blue cones are excited by the inputs. We also point out that the brightness-hue confound can make some experimental procedures give more accurate and stable results than others. In particular, the procedure used by Pokorny and Smith[1], in which subjects only need to judge whether the two fields differ, is better than other matching procedures in which subjects need to match the brightness of the two fields before judging whether they differ in hue.

The factors responsible for the low overall quantum efficiency of wavelength and other simple discriminations are unknown, but presumably they include photoreceptor inefficiencies, limits in the spatial and temporal integration (by the post-receptor neural mechanisms) of the photoreceptor responses, and neural noise. Any of these factors would tend to reduce overall quantum efficiency while preserving constant relative efficiency[4], [ 6].

**Figure 7:**
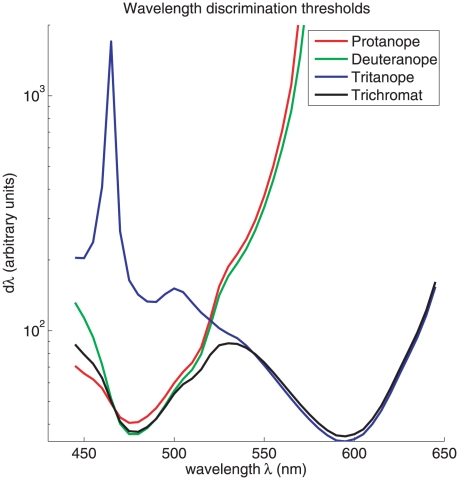
Theoretical preditions of the wavelength discrimination by dichromatics as compared to that by the trichromats. All these curves are by fixing input intensity 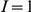, while using 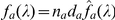 in which 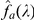 is normalized by Max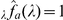, while 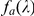 are no longer normalized by Max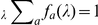. The values 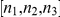 are 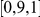, 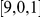, 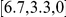, and 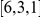 for protanopes, deuteranopes, tritanopes, and trichromats, respectively.

Our method in this paper can easily be applied to predict wavelength discrimination by dichromats. Fig. 7 shows that, compared with the trichromats, the protanopes and deuteranopes should have much larger thresholds in the long wavelength region, and the tritanopes should have much larger thresholds in the short wavelength region. These predictions seem to suggest that, for trichromats, wavelength discrimination is mediated by the protanopic/deuteranopic system at short wavelengths and on the tritanopic system at long wavelengths. These theoretical predictions are in line with known observations[25]. They are intuitively expected since color discrimination in the long wavelength region requires the combined activations of both L and M cones, while the S cones are essential for short wavelength discrimination since L and M cones are both only weakly active and co-vary considerably in that wavelength region. These qualitative predictions are insensitive to the actual cone densities used in our formula. These results are arrived at by assuming that the number of L/M cones in a protanope/deuteranope is the same as the total number of L and M cones in a trichromat (as suggested by data from[26]), and that the missing S cones in tritanopes are replaced proportionally by additional L and M cones so that the total number of cones is conserved. The predictions then follow naturally from equation (28) except to replace all summations over three cone types by the corresponding summations over two cone types. One caveat of these predictions is that the large threshold predictions, especially for the dichromats, should be taken as only qualitatively rather than quantitatively trustworthy. This is because our Fisher information formulation for discriminability is based on discriminating two stimuli very close to each other such that a Taylor expansion of log likelihood ratio is a suitable approximation. The suitability of this approximation breaks down when the two stimuli are very different from each other, when the discrimination threshold is too large. This issue has been raised by a previous work on tritanopia[27].

Our formulation of an ideal observer analysis for sensory feature discrimination under perceptual confound is general, and can be used in other sensory discriminations beyond our example case in this paper. More specifically, let a sensory world contain two feature dimensions, whose feature values are denoted by 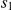 and 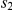 respectively, hence 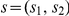. And suppose we have an experiment to find the minimum difference in feature 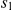 needed to distinguish a comparison input from a standard input, regardless of the feature 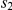 in the comparison input, analogous to the method of Pokorny and Smith[1]. Let 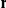 be the population neural responses to the sensory input 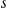 with probability 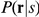. One can derive Fisher information matrix 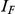 as in equation (17) with elements 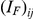; then equation (20) gives the discrimination threshold in 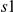 while feature 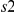 may present a perceptual confound.
